# Current Role of Intracoronary Imaging for Implementing Risk Stratification and Tailoring Culprit Lesion Treatment: A Narrative Review

**DOI:** 10.3390/jcm12103393

**Published:** 2023-05-10

**Authors:** Enrico Fabris, Elvin Kedhi, Monica Verdoia, Alfonso Ielasi, Maurizio Tespili, Giulio Guagliumi, Giuseppe De Luca

**Affiliations:** 1 Cardiothoracovascular Department, University of Trieste, 34100 Trieste, Italy; 2 Cardiology Division, Erasmus Hospital, Université libre de Bruxelles (ULB), 1050 Brussels, Belgium; 3 Department Medical, University of Silesia, 40-032 Katowice, Poland; 4 Division of Cardiology, Ospedale degli Infermi, ASL Biella, 13875 Biella, Italy; 5 Division of Cardiology, IRCCS Hospital Galeazzi-Sant’Ambrogio, 20161 Milan, Italy; 6 Division of Cardiology, AOU “Policlinico G. Martino”, and Department of Clinical and Experimental Medicine, University of Messina, 98100 Messina, Italy

**Keywords:** intracoronary imaging, OCT, IVUS, NIRS, culprit lesion, acute coronary syndrome, thin cap fibroatheroma

## Abstract

Our understanding of the pathophysiology of acute coronary syndrome and of the vascular biology of coronary atherosclerosis has made enormous progress with the implementation of intravascular imaging. Intravascular imaging contributes to overcoming the known limitations of coronary angiography and allows for the in vivo discrimination of plaque morphology giving insight into the underlying pathology of the disease process. The possibility of using intracoronary imaging to characterize lesion morphologies and correlate them with clinical presentations may influence the treatment of patients and improve risk stratification, offering the opportunity for tailored management. This review examines the current role of intravascular imaging and describes how intracoronary imaging represents a valuable tool for modern interventional cardiology in order to improve diagnostic accuracy and offer a tailored approach to the treatment of patients with coronary artery disease, especially in the acute setting.

## 1. Introduction

Coronary artery disease (CAD) is a leading cause of death worldwide, and acute coronary syndromes (ACS) are among the most frequent manifestations of ischemic heart disease [1,2]. Despite the wide application of an invasive strategy, some subsets of patients remain at high risk of subsequent cardiovascular events [3,4]. Therefore, large efforts have been done in the last decades to investigate new risk factors and to implement diagnosis and pharmacological and invasive treatment of ACS [5,6,7,8,9,10,11,12].

Coronary angiography has been used as the gold standard to evaluate the presence, location, and severity of coronary artery disease; however, it provides little or no information regarding plaque composition and biological activity. Intravascular ultrasound (IVUS), near-infrared spectroscopy (NIRS)-IVUS, and optical coherence tomography (OCT) are commercially available intracoronary imaging modalities that allow the visualization and measurements of the lumen, vessel and the evaluation of atherosclerotic plaques composition. 

The pathophysiological approach to ACS as well as the vascular biology of coronary atherosclerosis have made enormous progress with the implementation of intravascular imaging data over the past two decades. Indeed, intravascular imaging allows for the in vivo discrimination of plaque morphology in patients presenting with ACS with potentially important clinical implications. Indeed, in patients with type 1 myocardial infarction, intravascular imaging may detect three common underlying mechanisms of coronary thrombosis: plaque rupture (PR), plaque erosion (PE), or the presence of calcified nodules (CN), and the determination of these mechanisms may provide an advance towards the goal of a tailored therapy of different culprit lesions. Moreover, the extensive in vivo intracoronary imaging investigation supports the true existence of the so-called “vulnerable” plaques.

Therefore, this article aims to review the literature on the use of intravascular imaging to improve diagnostic accuracy and to offer a tailored approach to treatment for ACS patients. Moreover, we describe how the detection and quantification of plaque components are key to assessing the risk of plaque vulnerability.

## 2. Intracoronary Imaging Modalities

Optical coherence tomography (OCT) achieves very high-resolution image details through a light-based, near-infrared spectrum emitted from a single fiber optic wire, rotating at high speed when pulled back into the vessel. OCT is uniquely placed among the available imaging systems due to an exclusive axial and lateral resolution (10 and 70 μm) [13,14]. A main limitation of OCT is the penetration depth that decreases significantly in lipid plaques (0.2 mm), hampering efforts to assess plaque burden and measure the depth and volume of lipid pools. To image, OCT needs a blood-free field during the acquisition. The second generation of OCT systems (Fourier domain OCT) enabled rapid imaging of the coronary arteries without occlusive acquisition, and images of long segments can be acquired, maintaining good longitudinal resolution during short contrast injections [15].

IVUS utilizes ultrasound delivered to the vessel wall through a rotating catheter, pulled back into the vessel at a much lower speed compared to light-based OCT, also providing a much lower axial resolution (100 μm), IVUS can easily penetrate plaque up to 10 mm, but not calcium that blocks and shadows the ultrasound signal [16,17]. NIRS (Near InfraRed Spectroscopy) is a novel imaging modality based on the spectroscopic properties of the tissues, separated by absorbed and scattered infrared light at different intensities and wavelengths [18]. Given the different strengths and weaknesses existing in each imaging modality and the need to quantify multiple plaque components to assess the risk, the integration of multimodality imaging technologies in a single catheter has been recently attempted. The combination of NIRS with IVUS in a hybrid imaging catheter (NIRS-IVUS) allows accurate detection of lipids, displayed as a chemogram, with automatic quantification of the lipid core burden (LCB), coupled with all other features provided by IVUS, such as lumen size, plaque burden, and architecture [18]. Conversely, to achieve maximal resolution on high-risk lumen plaque features (e.g., thin cap fibroatheroma (TCFA), macrophages) with co-localization of deeper lipid core components, NIRS has been recently associated with OCT. Indeed, a critical measure of plaque stability is the fibrous cap thickness. The “in vivo” identification of TCFA finds great opportunities with OCT to screen high-risk plaques [19] as accurate thickness measurements are challenged by lipid attenuation and light scattered contour, high-quality OCT images and non-artifacts (TCFA-like images due to tangential catheter position) are required. Finally, also other determinants of vulnerable plaque like thrombus, macrophage infiltration, cholesterol crystals, microchannels and low-intensity areas can be assessed by OCT. 

In summary, OCT, with its higher resolution, is more suitable for close-up detail viewing and provides superior insight regarding the presence of vulnerable plaque features compared to IVUS (Table 1). Therefore, when the purpose is to search for features of vulnerable plaques, OCT should be preferred compared to IVUS as well as for searching for signs of plaque instability like the presence of thrombosis and detection of plaque erosion. Importantly IVUS-NIRS, unlike OCT, does not require image interpretation for the detection of LCBs and allows automatic quantification of LCBs without the need for manual image processing and thus may be used without extensive expertise for detecting lipid plaque. OCT, however, has less tissue penetration than IVUS, and its ability to visualize the external elastic lamina is impaired, particularly in large vessels and when the wall thickness is beyond the penetration depth [15,17]. In contrast, IVUS is very useful for vessel sizing in large and diseased segments and thus should be considered for accurate estimation of balloon and stent diameter in these cases. While the anatomy of the vessel can be considered a factor that may influence the choice of the modality of intracoronary imaging, OCT requires contrast injection, and thus, its use may be limited in patients with poor renal function, and in those cases, IVUS should be considered. Both techniques can be used to optimize stent implantation and guide optimal stent expansion [20]; however, edge dissection, stent malapposition and tissue protrusion are better defined with OCT [21]. 

To date, there is no evidence of the superiority of one intravascular imaging technique compared to the other in clinical outcomes, and therefore they should be considered complementary tools. However, as the guidance with intravascular imaging improves the clinical outcomes of patients undergoing percutaneous coronary intervention [22], interventionalists should become familiar with at least one modality based on individual preference, availability and cost.

## 3. Evaluation of Pathophysiology of Culprit Lesions

ACS and type 1 myocardial infarction (MI) are caused by thrombosis developing on a culprit coronary atherosclerotic plaque and critical reduction in blood flow. The exposure of the subendothelial structures and the subsequent activation of platelets by contact with collagen are responsible for thrombosis with the formation of an occlusive or sub-occlusive thrombus with patients usually presenting with ST-elevation MI (STEMI) or non-ST-elevation MI (NSTEMI), respectively. 

Intravascular imaging has sharpened our ability to characterize the culprit lesions of ACS in living patients. There are three common underlying mechanisms of coronary thrombosis clinically manifesting as ACS: plaque rupture (PR), plaque erosion (PE) and calcified nodule (CN).

**Figure 1:**
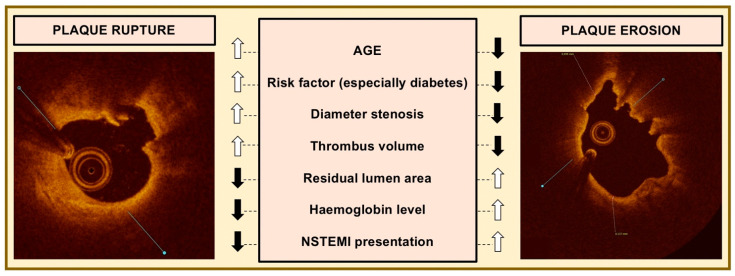
Common features in plaque rupture vs. plaque erosion. Legend: up arrows indicate higher frequency/values while down arrows indicate lower frequency/values compared to the other plaque type.

Each of these mechanisms tends to have specific plaque features (Figure 1), and the determination of these mechanisms may provide an advance toward the goal of a tailored therapy of different culprit lesions.

### 3.1. Plaque Rupture

PR, followed by intracoronary thrombus formation, is recognized as the most common pathophysiological mechanism in ACS. PR usually occurs in inflamed TCFA, which presents a fibrous cap disruption. Disruption of TCFA directly exposes the underlying necrotic core to the blood with its coagulation factors, and the thrombogenic material residing within the plaque promotes thrombosis. PR is characterized by major distortion of the vessel architecture with a positive remodeling, large necrotic core, fibrous cap disruption, intraplaque cavity, large thrombus burden and a small residual lumen area.

In the infarct-related/target lesions, PR and intracoronary thrombus are frequently observed, and compared with lesions in patients without MI, fibrous cap thickness is significantly thinner, and the frequency of OCT-derived TCFA is significantly greater [23]. Importantly non-culprit lesions in patients with ACS have more vulnerable plaque characteristics compared with those with non-ACS [24]. 

This could also explain why, in STEMI, complete revascularization of “stable” plaque is associated with a reduction in CV mortality compared with culprit-lesion-only PCI [25,26]. Indeed, ACS presentation may be considered a clinical marker that represents the patient’s biological propensity to develop vulnerable plaques (i.e., thinner cap and a higher rate of TCFA), which expose the patients to a higher rate of MACE if left untreated. The COMPLETE Trial OCT sub-study showed a high prevalence of patients with at least one TFCA lesion (47%) in patients with STEMI [27]. Conversely, PCI of “stable” plaque in patients with stable CAD reduces angina frequency and improves the quality of life but does not improve myocardial infarction and cardiovascular mortality [28]. Moreover, while it is still unclear why some plaque lead to STEMI, whereas others cause NSTEMI, it has been shown that plaque morphologies in the culprit lesion might affect the clinical presentations in patients with ACS. Ino et al. compared culprit lesion morphologies between STEMI and NSTEACS using OCT and found that PR, TCFA and red thrombus were more often seen in STEMI [29].

### 3.2. Plaque Erosion

The second most common underlying substrate for ACS is PE. Multiple studies have reported the prevalence of plaque erosion in approximately 20–40% of patients with ACS [30,31]. PE is characterized by a luminal thrombus without evidence of fibrous cap disruption but the presence of endothelial denudation [32]. Even though the endothelial monolayer cannot be visualized directly by an intravascular imaging technique, OCT may identify the presence of a thrombus overlying a plaque that has an intact fibrous cap [15]. In MI patients, plaque erosion can be identified more frequently by OCT in comparison with IVUS [33]. Definite erosion is identified by the presence of a luminal thrombus overlying an intact plaque, while probable erosion is defined as luminal surface irregularity at the culprit lesion in the absence of a clear thrombus. Interestingly, thrombi that complicate superficial erosion seem more platelet-rich than the fibrinous clots precipitated by PR.

We have limited knowledge of the mechanisms of superficial erosion. However, these OCT-based intact fibrous cap plaques are less likely to be associated with positive remodeling and lipid core and are more frequently present in smokers or females [34]. The prevalence of PE is also related to the clinical context, with PR being more frequent in patients with STEMI compared with NSTE-ACS in in vivo studies [29]. Indeed, an OCT-based study of patients presenting with ACS showed that patients with OCT-erosion less frequently present with STEMI than those with PR, and NSTEMI was the predominant presentation for the patients with OCT-erosion [30]

In NSTEMI, five independent parameters have been associated with plaque erosion: age < 68 years, anterior ischemia, no diabetes mellitus, hemoglobin > 15.0 g/dL and normal renal function. When all these five parameters were present, the probability of plaque erosion increased to 73.1% [35]. Patients with PE also have less frequent multivessel disease involvement and lower Syntax and Gensini scores compared to patients with PR.

In a recent study enrolling a total of 822 STEMI [36] patients suitable for culprit lesion evaluation by OCT, PR accounted for 69% and PE for 25%. Interestingly, patients with PE had lower total cholesterol and LDL levels as compared with ruptures and also less diabetes, hypertension, dyslipidemia and chronic kidney disease. Age < 50 years, nearby bifurcation and absence of dyslipidemia were predictive of PE. PE lesions had a lower percentage of stenosis and a larger minimal lumen area as compared with PR. Moreover, TCFA was less frequently observed in erosion cases than in ruptures [36]. 

Furthermore, in STEMI patients, PE, compared with PR, was associated with higher rates of patent IRA at first angiography [37] and a lower incidence of the no-reflow phenomenon after PCI [38]. A recent study that evaluated 1113 patients with ACS who underwent OCT also reported possible seasonal variations in the underlying pathobiology of ACS, with a proportion of PR being highest in winter, whereas that of PE was highest in summer [39]. Outbreaks of infectious diseases during the winter months, especially influenza and other respiratory infections, can contribute to systemic inflammation, which can contribute to plaque rupture. Moreover, there is some seasonal variation in hypertension, with higher blood pressure in winter than in summer. Hypertension, influencing shear stress, may be a potential mechanical trigger for plaque rupture.

In addition, the molecular physiology underlying PE seems to have some important features as a higher expression of toll-like receptor 2, neutrophil, myeloperoxidase and hyaluronidase [40].

These studies suggest that PE may have different pathophysiology and distinct clinical characteristics and that “non-traditional” factors such as local flow disturbance and high endothelial shear stress, which may promote a low-level inflammatory activation of the luminal endothelial cells and cell desquamation, could play an important role.

### 3.3. Calcified Nodule

Calcified nodules (CNs) are the least common cause of coronary thrombosis and usually occur in a coronary segment with extensive calcification. Its prevalence is about 2–8% of patients with ACS and is generally more prevalent in older men and in patients with tortuous coronary arteries, diabetes mellitus, or chronic renal failure [41].

Thrombosis occurs when nodular calcification breaches the overlying fibrous cap, thus a luminal surface that is disrupted by nodules of dense calcium. On IVUS/OCT, CNs appear as protruding calcific masses with irregular surface and dorsal shadowing and, by definition, have an overlying thrombus. Lesions with CNs are associated with a small thrombus burden and small or no necrotic core.

## 4. Tailored Therapy Based on Imaging Findings and Underlying Plaque Morphology

The premise of precision medicine is “personalized” treatment, which means an individualized approach to treatment, taking into account inter-individual variations. Considering the potential different pathobiology of PR, PE and CN, the direct visualization of these different plaque morphologies may provide a glimpse into the vascular biology of the disease, and this characterization might aid in the derivation of tailored treatment strategies.

Patients presenting with ACS and candidates for percutaneous coronary intervention are uniformly treated with stenting regardless of the underlying pathology. However, this one-size-fits-all approach might be modulated and personalized in some individuals. Interestingly, patients with ACS presenting with PR as a culprit lesion have a worse prognosis compared with patients with PE, and this should be taken into account in risk stratification and management [42]. Moreover, considering that PR is associated with higher plaque burden and higher vessel stenosis compared to PE and that the necrotic core in PR may act as a persistent stimulus for thrombosis and re-occlusion, in cases of PR, stent implantation may serve to seal the plaque. Conversely, PE is often devoid of a necrotic core, and if present, this does not communicate with the lumen because of the presence of an intact fibrous cap. Therefore, in ACS with non-obstructive lesions and PE, an alternative treatment strategy focusing on anti-thrombotic therapies only rather than stenting may deserve consideration. Indeed, treatment with anti-platelet drugs might allow healing of the denudated endothelial layer without the need for stenting and thus avoiding the long-term risk of stent failure as stent thrombosis and in-stent restenosis.

Prati et al. [43] have challenged the dogma of stenting all STEMI patients, showing that STEMI patients with OCT-detected PE remained asymptomatic after more than 2 years of clinical follow-up, regardless of stent implantation. Another proof-of-concept non-randomized, uncontrolled, prospective study, the recent EROSION (Effective Anti-Thrombotic Therapy Without Stenting: Intravascular Optical Coherence Tomography-Based Management in Plaque Erosion) study [44], showed that in 55 ACS patients with OCT-detected PE, a residual vessel stenosis < 70% and a TIMI flow grade III on angiography, a conservative treatment with potent antithrombotic therapy (i.e., aspirin and ticagrelor, and heparin ± glycoprotein IIb/IIIa inhibitor) without stenting may be an alternative strategy, with 92.5% of patients free of a major adverse cardiovascular event for ≤1 year [45]; however, the study lacked an arm of prompt stenting. At four-year follow-up, findings reconfirmed the safety of an anti-thrombotic therapy without stenting (all patients were free from hard endpoints: death, myocardial infarction, stroke, bypass surgery or heart failure), although approximately 20% of patients underwent target lesion revascularization [46]. 

In another single-center, single-arm, unblinded prospective study including a total of 252 (55 patients were from the EROSION study [44]) patients aged ≥60 years, percentage of area stenosis ≥63.5% and thrombus burden ≥18.5% were the best cut-off values of predictors of major adverse cardiovascular events (MACE) in patients with ACS caused by PE with a non-stent strategy [47].

The randomized EROSION III trial [48] enrolled STEMI patients without obstructive stenosis and compared OCT vs. angiographic guidance in optimizing the reperfusion strategy. The study revealed that PR, PE and CN were present in 66%, 29% and ~5%, respectively and showed that OCT guidance could reduce the rate of stent implantation during primary PCI (stent implantation occurred in 43.8% of patients randomized to OCT guidance and 58.8% of patients undergoing angiographic guidance, *p* = 0.024), with the MACE similar in both groups, although the study was underpowered for clinical outcome assessment. 

Identifying the optimal treatment strategy for treating PE remains a current challenge. Even though the development of personalized treatments contrasting the one-size-fits-all kind of approach is advisable and avoiding stent may be an option in selected cases, the limited number of subjects included in dedicated studies does not allow for drawing definite conclusions and limit the applicability in the real world. Moreover, in patients without stenting, the optimal duration of treatment and drug choice remains unclear. Larger randomized clinical trials comparing standardized medical therapy to revascularization could improve our ability to tailor personalized approaches based on plaque morphologies. However, a well-powered trial would require a very large population to show evidence of the superiority of imaging guidance based on plaque morphology.

In the meantime, a continuous effort should be placed on mitigating risk factors and evaluating atherosclerotic burden, which remains an important metric of risk stratification of patients. 

Indeed, a change in coronary artery plaque characteristics and the increasing incidence of PE rather than PR may derive from better control of cardiovascular risk factors and the increasing use of statin and the subsequent reduction in lipid levels and vascular inflammation [49]. Moreover, changes in size and plaque composition over time can be evaluated by intravascular imaging and may help to understand the impact of treatment [50].

## 5. Searching for the Culprit

**Figure 2:**
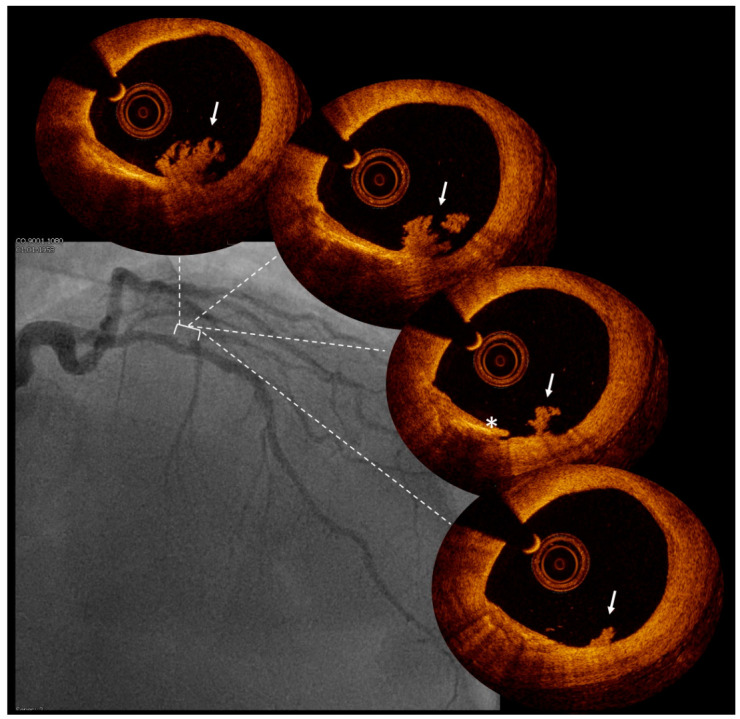
Intracoronary thrombus detection by optical coherence tomography. Legend: coronary angiography of a patient presenting with minor anterior non-ST elevation myocardial infarction without obstructive coronary artery disease. Optical coherence tomography showed the presence of a thrombus (arrow) near a small dissection (*), giving proof of an acute coronary event.

**Figure 3:**
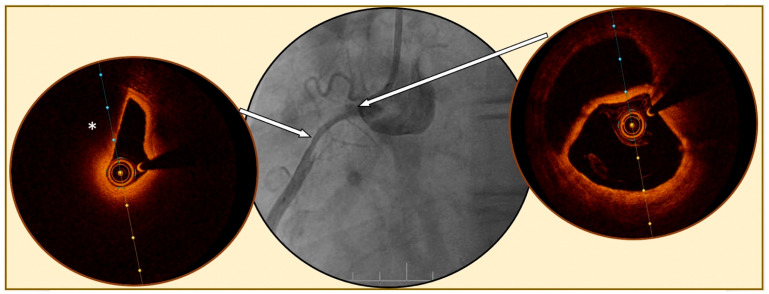
Spontaneous coronary artery dissection evaluated by optical coherence tomography. Legend: Angiography of a right coronary artery (**central figure**) showing a diffuse tabular narrowing highly suspected for spontaneous coronary artery dissection. Optical coherence tomography (OCT) images confirm the diagnosis of a large dissection, with the presence of a false double lumen (**right figure**), at the beginning of the dissection (right arrow) and compression of the true lumen (**left figure**) by a diffuse circular hematoma (*) in the mid lesion (left arrow).

A clinically relevant proportion of patients presenting with ACS have non-obstructive coronary artery disease on coronary angiography, and this may pose significant challenges in diagnosis and treatment [51]. Complementary invasive imaging enhances the diagnostic accuracy of culprit lesion detection, and a common indication in clinical practice for using OCT is to search for the culprit lesion when angiographic findings remain inconclusive. Indeed, OCT may help to understand the mechanisms potentially underlying MI in MI with non-obstructive coronary artery disease (MINOCA) patients, for example, by detecting the presence of a thrombus giving proof of an acute coronary event [52,53,54] (Figure 2). In a recent study [55] including 190 MINOCA patients, OCT identified a cause in 61.1% of MINOCA. Moreover, while PE, PR, and CN are possible causes of atherosclerotic MINOCA, non–atherosclerotic causes of SCA include spontaneous coronary artery dissections, which can be evaluated with intracoronary imaging (Figure 3), coronary spasm and unclassified causes. Importantly, MACE seems to be significantly different between atherosclerotic and non–atherosclerotic lesions, with a higher event rate in atherosclerotic lesions, highlighting that atherosclerotic-MINOCA represents an important and distinct MINOCA subset [55]. The next important step for MINOCA is to demonstrate the impact of personalized treatments. The PROMISE study [56], a randomized, multicenter, prospective, open-label, superiority, phase IV trial, will compare a “precision-medicine approach” versus a “standard of care approach” in MINOCA patients to test the prognostic value of a targeted treatment approach based on the identification of the underlying disease using of a comprehensive diagnostic workup, which includes OCT assessment.

## 6. The Vulnerable Plaques: Precursors of Coronary Events?

The concept of high-risk plaques emerged decades ago, opening a new era of research aimed at advancing our knowledge of precursors of adverse cardiac events. Indeed, pathological studies have primarily identified anatomical markers of coronary plaques underlying major cardiac events [57], some features now under extensive in vivo investigation with high-resolution intravascular imaging to make predictable the so-called “vulnerable” plaques [19,58]. These plaques are characterized by a large lipid core and a thin fibrous cap (<60–75 μm), often with signs of ongoing inflammation, such as the presence of macrophages and cholesterol clefts. Interestingly, these lesions are not necessarily associated with severe coronary artery stenosis [59]. Certainly, ACS often arise from rupture and thrombosis of lipid-rich atherosclerotic plaques, which may be mild to moderate on angiography and are non-flow-limiting. Therefore, the impact of intracoronary imaging becomes even more pivotal, allowing the quantification and complete featuring of the plaque and the detection of those factors promoting its destabilization. Several trials, as summarized in Table 2, have so far suggested that plaque burden (defined as the total amount of plaque normalized to the patient’s individual coronary vessel volume) and composition can play a major prognostic role, in addition to residual vessel lumen (minimal lumen area). Identification of macrophages, TCFA or lipid core plaque (LCP), based on the distinction of spectral features differentiating cholesterol, cells, necrotic material and collagen, has been provided with recent technological acquirements. The dichotomous definition of coronary artery disease as “obstructive or flow limiting” and “nonobstructive” seems inaccurate for identifying truly high-risk patients [60,61,62]. In effect, true weight on the risk of future CV events provided by the presence of flow-limiting stenosis per se or due to plaque characteristics, which may be present also in non-obstructive CAD, is still uncertain.

The PROSPECT study [63] showed for the first time that coronary plaques with TCFA and a large plaque burden were associated with adverse cardiac events. Moreover, the ability of IVUS-NIRS to detect lesions at high-risk for future adverse events has been proven by the lipid-rich plaque (LRP) study [64]. The PROSPECT II study [65], enrolling patients with recent myocardial infarction and non-culprit non-flow limiting lesions with at least 40% plaque burden, confirmed and extended the previous results of the LRP study. The PROSPECT II showed that adverse cardiac events occurred in 13% of patients within 4 years, but with 8% arising from benign-appearing and non-flow-limiting lesions at baseline [65], therefore highlighting the incremental value of identifying high lipid content with NIRS in lesions with a large plaque burden identified by IVUS.

While NIRS-IVUS made a step forward in detecting vulnerable plaque with large lipid cores, the low resolution of IVUS does not permit for reliably measuring the atheroma plaque cap thickness. The relevance of this feature is paramount; indeed, while lipid-rich plaques are common, they are less likely to be associated with adverse cardiac events in the absence of TCFA [66].

Lipid-rich plaque is defined as TCFA when the thinnest part of the atheroma cap measures ≤65 μm on OCT assessment. However, the exact cut-off value for the fibrous cap or arc of the lipid pool for the identification of an OCT-TCFA is still debated. The recent CLIMA study [58] identified high-risk plaque features as MLA <3.5 mm^2^, fibrous cap thickness <75 μm, lipid arc circumferential extension >180° and presence of macrophages. The concurrent presence of these four OCT criteria in the same plaque was observed in 18.9% of patients experiencing MACE at one year and was independently associated with events.

The natural history of OCT-detected TCFA lesions in diabetes mellitus (DM) patients with negative fractional flow reserve (FFR) lesions was assessed in the COMBINE study [19,67], which enrolled 550 DM patients undergoing coronary angiography for either ACS or stable coronary disease and with at least one lesion with stenosis between 40–80%. In patients with negative FFR, the incidence of target lesion-related MACE was high in patients with TCFA-positive lesions, who had an almost five-fold higher incidence of MACE versus TCFA-negative patients [19]. The detected vulnerable plaques by OCT represented up to a quarter of angiographically intermediate FFR-negative lesions but were responsible for more than 80% of future adverse events. Within TCFA-carrying patients, a smaller minimal lumen area (MLA), lower FFR values and TCFA location adjacent to a healed plaque were associated with future events [68]. Importantly, this increased risk of adverse events persisted during long-term follow-up [69].

These current findings support the true existence of vulnerable plaques and the use of intravascular imaging modalities to detect them and to guide preventive and individualized treatment among patients with CAD. Alternatively, use of a non-invasive imaging tool, such as computer tomography angiography, could be used to search for similar information. We have learned that high-risk plaques are really consistently associated with follow-up clinical events, and this lays the groundwork for powered randomized trials for testing promising systemic and focal treatments, including pharmacotherapy to sequester these plaques or through mechanical plaque pacification with intracoronary devices. 

## 7. Future Perspectives

The most frequent indication for intravascular imaging is stent-optimization [70] in order to maximize the stent expansion and correct strut malapposition, but intravascular imaging is also frequently used for guiding stent implantation and to plan strategy of intervention in different clinical scenarios [71,72,73] Indeed, intravascular imaging may help to decide whether or not revascularize, guiding the appropriate procedural techniques and help to select the appropriate stent diameter and length. The use of intravascular imaging has been associated with improved PCI outcomes in both randomized trials and registries [22] and seems particularly important to use it when treating prognostic segments as the left main [74]. Moreover, the possibility of using intracoronary imaging to make in vivo diagnosis of plaque biology, characterizing lesion morphologies and correlating them with clinical presentations may influence the treatment of patients with ACS and improve risk stratification, offering the opportunity for tailored management. Appropriate education on imaging interpretation represents a future challenge for intravascular imaging clinical implementation.

The integration of multimodality imaging technologies in a single catheter as a hybrid IVUS-OCT [75] or OCT-NIRS [76] could provide a comprehensive assessment of the coronary vasculature providing a complementary combination of OCT for plaque structure with NIRS for plaque composition. Advances in technology and the integration of artificial intelligence and machine learning have the potential to enhance the interpretation of images reducing interobserver variability. 

Future studies will assess the value of a tailored therapeutic approach of combined OCT-guided focal percutaneous treatment and optimal medical therapy in order to prevent adverse hard cardiac events. The COMBINE INTERVENE (NCT05333068) and PREVENT (NCT02316886) trials will focus on non-ischemic (FFR > 0.75) vulnerable plaques to compare revascularization versus the medical treatment and the INTERCLIMA study (NCT050227984) will compare a functional versus OCT-guided stenting strategy.

## 8. Conclusions

Intracoronary imaging represents a relevant tool in the management of patients with ACS that clearly contributes to overcoming the known limitations of coronary angiography. It provides detailed information on the atherosclerotic plaque giving insight into the underlying pathology of the disease process and has the potential to guide proper and tailored treatment in an acute setting. Moreover, recent findings support the use of intravascular imaging modalities to detect vulnerable plaque to improve risk stratification and hopefully guide in the next future preventive and individualized treatment. A larger utilization of these modalities, future dedicated randomized studies and continuous technological advances might certainly determine a major shift in the management of patients with CAD.

## Figures and Tables

**Table 1:** Differences between intravascular imaging techniques.

	IVUS	OCT
**Technical features**		
Waves	Ultrasound	Near-infrared light
Axial resolution (μm)	100–150	10–20
Lateral resolution (μm)	150–300	20–70
Tissue penetration (mm)	4–10	0.5–2
Need for blood clearance	no	yes
**Lesion evaluation**		
Ostial Left main	••	-
Large vessel diameter	••	•
Plaque burden	••	•
Lipid Core	•	••
Calcium depth	••	•••
Thrombus detection	•	•••
TCFA	-	•••
Macrophage infiltration	-	•••
Cholesterol crystals	-	•••
Microchannels	-	•••
Ease of image interpretation	•	•••
**Acute stenting evaluation**		
Stent expansion	•••	•••
Edge dissection	•	•••
Stent malapposition	••	•••
Tissue protrusion	•	•••

••• Excellent capability; •• Good capability; • sufficient capability; - insufficient capability; TCFA: thin cap fibroatheroma; IVUS: Intravascular ultrasound; OCT: Optical coherence tomography.

**Table 2:** Studies supporting the existence of vulnerable plaques.

Year	Study	Number of Patients	Lesions Studied	Intravascular Imaging	Predictors of MACE
2011	PROSPECT [63]	697	Non-culprit lesions in ACS pts	IVUS	- **Plaque burden ≥ 70%** (HR, 5.03; 95% CI, 2.51–10.11) - **MLA ≤ of 4.0 mm^2^** (HR 3.21; 95% CI, 1.61–6.42) - **Presence of TCFA** (HR 3.35; 95% CI, 1.77–6.36)
2019	LIPID-RICH PLAQUE [64]	1552	Non-culprit lesions in pts with suspected CAD	NIRS-IVUS	Segments with **max LCBI4 mm > 400** (plaque level adjusted HR 3.39, 95% CI 1.85–6.20)
2020	CLIMA [58]	1060	Left anterior descending lesion in ACS and stable angina pts	OCT	- **MLA < 3.5 mm2** (HR 2.1, 95% CI 1.1–4.0) - **FCT < 75 µm** (HR 4.7, 95% CI 2.4–9.0) - **Lipid arc > 180°** (HR 2.4, 95% CI 1.2–4.8) - **Macrophages** (HR 2.7, 95% CI 1.2–6.1)
2021	PROSPECT II [65]	898	Non-flow-limiting non-culprit lesions in pts with previous MI	NIRS-IVUS	- **Max LCBI4 mm ≥ 324.7** (Lesion-level OR 7.83,95% CI 4.12–14.89) - **Plaque burden ≥ 70%** (Lesion-level OR 12.94, 95% CI 6.36–26.32) - **MLA ≤ 4·0 mm^2^** (Lesion-level OR 4.97,95% CI 2.59–9.53)
2021	COMBINE [19]	550	FFR negative non-culprit lesions in diabetic pts	OCT	**Presence of TCFA** (HR 4.65; 95% Cl, 1.99–10.89)

ACS denotes acute coronary syndrome; FFR fractional flow reserve, FCT fibrous cap thickness, MaxLCBI4 mm maximum 4 mm Lipid Core Burden Index, MACE major cardiovascular events, MLA minimal luminal area, MI myocardial infarction, Pts patients, TCFA thin-cap fibroatheroma. OR = Odds ratio, HR = hazard ratio, CI = confidence interval.

## Data Availability

Not applicable.
